# BiP, GRP94, calreticulin and calnexin contribute to development of the notochord in medaka fish

**DOI:** 10.1247/csf.25009

**Published:** 2025-05-13

**Authors:** Serina Kita, Tokiro Ishikawa, Kazutoshi Mori

**Affiliations:** 1 Department of Biophysics, Graduate School of Science, Kyoto University, Kyoto 606-8502, Japan; 2 Kyoto University Institute for Advanced Study, Kyoto 606-8501, Japan

**Keywords:** endoplasmic reticulum, protein folding, molecular chaperone, collagen, glycoprotein

## Abstract

The accumulation of unfolded or misfolded proteins in the endoplasmic reticulum (ER) activates the unfolded protein response (UPR) to maintain the homeostasis of the ER. The UPR consists of the IRE1, PERK and ATF6 pathways in vertebrates. Knockout of the IRE1 and PERK pathways causes defects in liver and pancreatic β cells, respectively, in mice, whereas knockout of the ATF6 pathway causes very early embryonic lethality in mice and medaka fish, a vertebrate model organism. We previously showed that ATF6 knockout in medaka causes a defect in the development of the notochord—the notochord becomes shorter—but that transient overexpression of the ER chaperone BiP via microinjection of BiP mRNA into one-cell stage embryos of these ATF6 knockout rescues this defect. Here, we microinjected mRNA encoding various ER chaperones and found that GRP94, calreticulin and calnexin also partially rescued this defect. Thus, BiP/GRP94 and calreticulin/calnexin greatly contribute to the development of the notochord by controlling the quality of collagens and *N*-glycosylated proteins (such as laminin and fibrillin), respectively, which have been confirmed necessary for the formation of the notochord in zebrafish.

## Introduction

Homeostasis of the endoplasmic reticulum (ER), where newly synthesized secretory and transmembrane proteins destined for the secretory pathway are folded and assembled, is challenged by a variety of physiological and pathological conditions which are collectively termed ER stress. ER stress is characterized by the accumulation of unfolded or misfolded proteins in the ER. It is sensed by the transmembrane protein Ire1 in yeast and by three types of transmembrane proteins, IRE1, PERK and ATF6, in vertebrates. Each of these four proteins triggers a unique signaling cascade to maintain the homeostasis of the ER (Mori, 2009). The overall effect of these activities is termed the unfolded protein response (UPR).

The UPR consists of translational and transcriptional programs coupled with intracellular signaling from the ER to the nucleus. The transcription factors downstream of IRE1 and PERK are known to be XBP1 and ATF4, respectively, in metazoans (Harding *et al.*, 2000; Yoshida *et al.*, 2001). The importance of the UPR has been demonstrated by constructing and analyzing knockout (KO) mice deficient in UPR mediators. For example, XBP1-KO mice exhibit embryonic lethality due to poor development of the liver, which is responsible for hematopoiesis during the mouse embryonic period (Reimold *et al.*, 2000). In contrast, PERK-KO mice develop diabetes after birth due to misfolded insulin-mediated proteotoxicity, which results in the apoptosis of pancreatic β cells (Harding *et al.*, 2001).

ATF6, consisting of ubiquitously expressed ATF6α and ATF6β in mice and in medaka fish, a vertebrate model organism, is activated by ER stress-induced proteolysis, resulting in production of the active and nuclear form of ATF6α/β, designated ATF6α/β(N). ATF6α/β(N) adjusts the levels of ER-localized molecular chaperones and folding enzymes (ER chaperones hereafter) by transcriptional induction in the nucleus in accordance with increased demands under ER stress, which in turn leads to the refolding of unfolded or misfolded proteins accumulated in the ER (Adachi *et al.*, 2008; Mori, 2003). ATF6α-KO and ATF6β-KO mice as well as ATF6α-KO and ATF6β-KO medaka exhibit no obvious phenotype under normal growing conditions, whereas ATF6α- and ATF6β-double KO (DKO) causes embryonic lethality in both mice and medaka (Ishikawa *et al.*, 2013; Yamamoto *et al.*, 2007). Because we could not obtain viable ATF6α/β-DKO embryos even at embryonic day 8.5 in viviparous mice, we could not determine why ATF6α/β-DKO mice die before birth. By taking advantage of oviparity, however, we found that notochord development was severely impaired in ATF6α/β-DKO medaka (Ishikawa *et al.*, 2013).

The notochord functions as the body axis before formation of the vertebra (Stemple, 2005). Notochord cells synthesize and secrete large amounts of extracellular matrix proteins such as type VIII collagen, This constitutes physiological ER stress and activates ATF6α/β to ensure the quality of these proteins (Ishikawa *et al.*, 2013). With the aid of induced ER chaperones, disk-like notochord cells smoothly align and the notochord extends to the tip of the tail. In the absence of ATF6α/β, however, the levels of mRNAs encoding various ER chaperones, namely BiP (Hsp70-type chaperone), GRP94 (Hsp90-type chaperone), calnexin (CNX, lectin-type transmembrane chaperone), calreticulin (CRT, lectin-type soluble chaperone) 1, CRT2, CRT3, and protein disulfide isomerase (PDI) are decreased. Accordingly, the alignment of notochord cells becomes disorganized and the extension of the notochord stops in the middle of the tail (Ishikawa *et al.*, 2013).

Importantly, we previously showed that microinjection of BiP mRNA into one-cell stage embryos of ATF6α/β-DKO medaka for transient overexpression rescued the phenotype; the notochord became longer and notochord cells showed smoother alignment (Ishikawa *et al.*, 2013). Here, we examined the effect of microinjection of mRNA encoding other ER chaperones on the length of the notochord.

## Results

### Design of rescue experiments

**Fig. 1:**
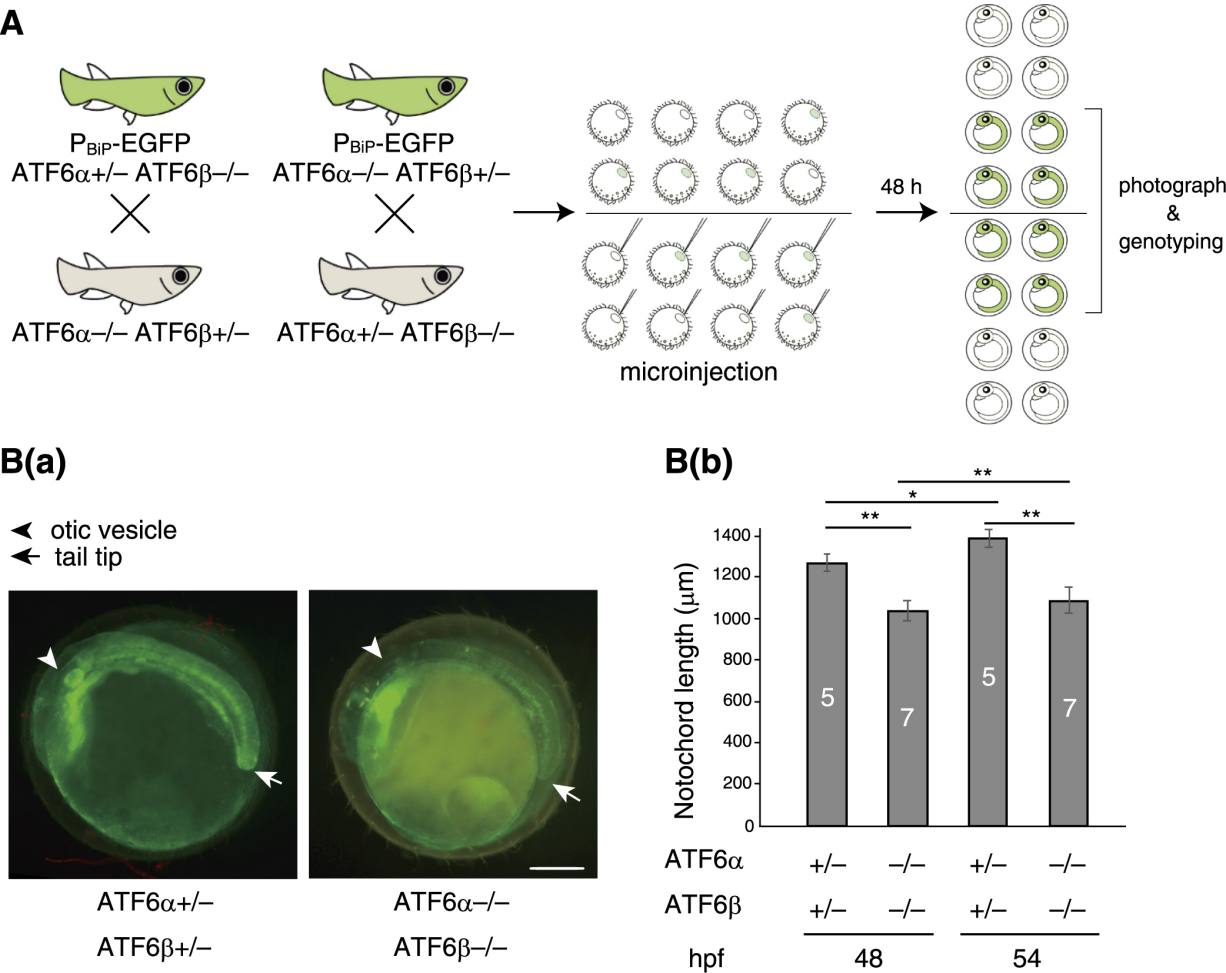
Design of rescue experiments (A) Experimental procedure. (B) Effect of ATF6α/β-DKO on length of the notochord. Scale bar; 250 μm. This experiment was conducted once with n number indicated in each column.

We measured the length of the notochord in medaka carrying P_BiP_-EGFP in its genome (expression of EGFP is under the control of the BiP promoter), because the notochord in addition to the brain and otic vesicle is fluorescently brightened due to the occurrence of physiological ER stress (Ishikawa *et al.*, 2013). Male ATF6α+/– ATF6β–/– medaka carrying P_BiP_-EGFP were crossed with female ATF6α–/– ATF6β+/– medaka, and male ATF6α–/– ATF6β+/– medaka carrying P_BiP_-EGFP were crossed with female ATF6α+/– ATF6β–/– medaka. Forty-eight and fifty-four hours after microinjection of ER chaperone mRNA into the obtained embryos, injected and non-injected embryos reaching stage 24 (heartbeat starts) ~ 25 (blood begins circulation) and stage 26 (guanophores develop), respectively, were photographed and genotyped (Fig. 1A). Although fluorescence emitted from the notochord in ATF6α/β-DKO embryos became much weaker compared with that in ATF6α/β-hetero embryos due to the absence of ATF6α/β-mediated activation of BiP promoter, we could easily identify the position of the otic vesicle by fluorescence and were able to determine the length from the otic vesicle to the tail tip precisely [Fig. 1B(a)]. The notochord in non-injected ATF6α/β-DKO embryos was significantly shorter than that in non-injected ATF6α/β-hetero embryos at both 48 and 54 hours post-fertilization (hpf) [Fig. 1B(b)].

### Molecular cloning of various medaka ER chaperones

**Fig. 2:**
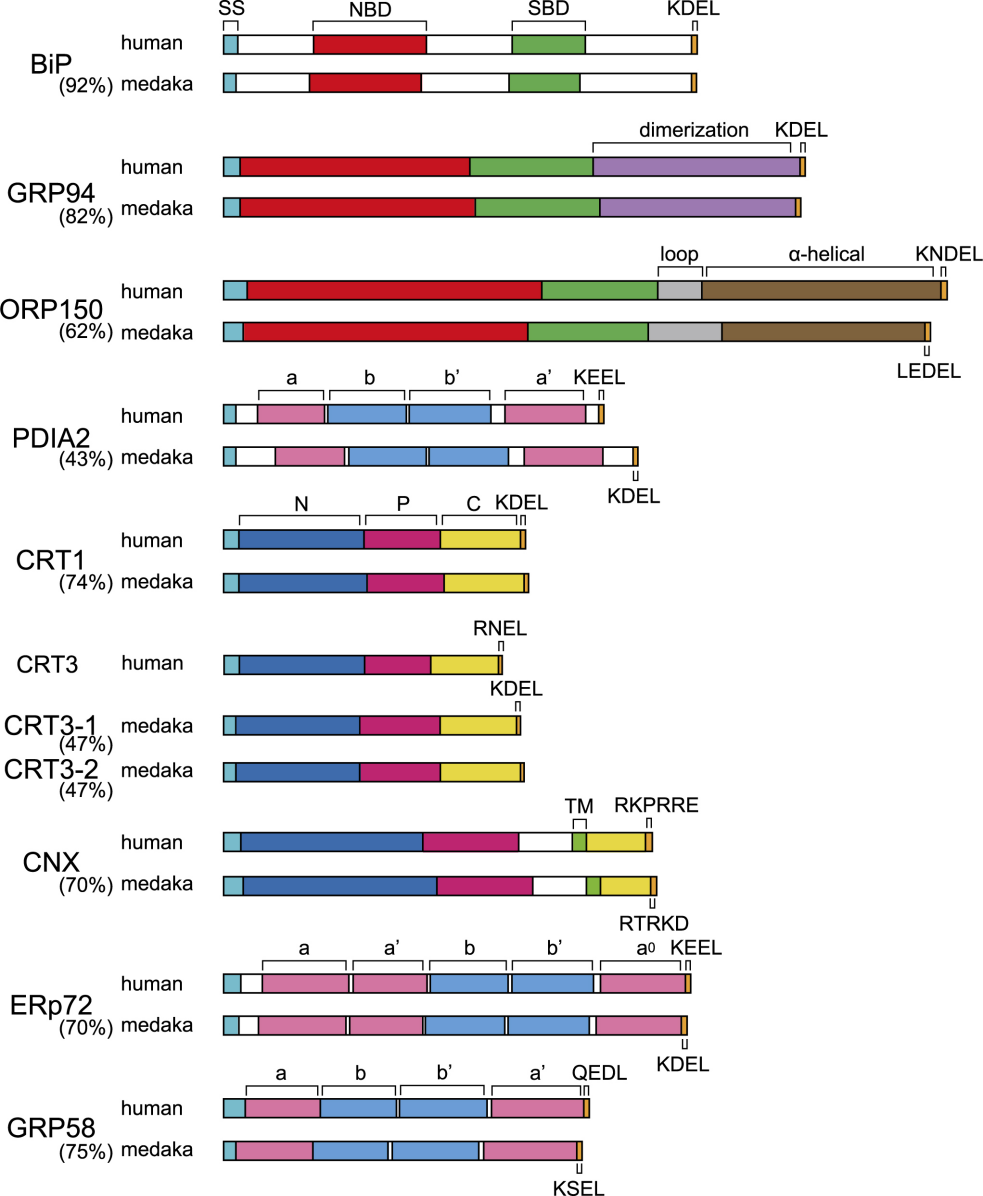
Domain structures of various ER chaperones in human and medaka Percentages in parentheses below the protein name indicate the identity between human and medaka proteins. SS, signal sequence; NBD, nucleotide binding domain; SBD, substrate binding domain. 4~5 C-terminal amino acids (each shown by one uppercase letter) of soluble ER chaperones function as an ER-retention signal. a, a' and a^0^ denote catalytically active thioredoxin-like domains containing a C-X-X-C motif, whereas b and b' denote catalytically inactive thioredoxin-like domains. N, P, and C denote N-terminal, proline-rich, and C-terminal domains, respectively. TM denotes a transmembrane domain.

We have cloned cDNA encoding GRP94, ORP150, PDIA2, CRTs, CNX, ERp72 and GRP58 (ERp57) from a medaka cDNA library using the information in the Ensembl Genome Browser (https://www.ensembl.org/Oryzias_latipes/Info/Index). Their domain structures are shown in Fig. 2. CRT2 was identified as a novel CRT1 isoform in human and mouse (Persson *et al.*, 2002). CRT2 is somehow registered as CRT3 in the Ensembl Genome Browser. Medaka and zebrafish have two CRT3 paralogues, CRT3-1/CRT3-2 and calr3b/calr3a, respectively. Although human CRT2 was reported to be expressed only in testis (Persson *et al.*, 2002), zebrafish calr3a was shown to be expressed in several tissues and most strongly in liver (Thakur *et al.*, 2011).

### Effect of transient overexpression of various ER chaperones

**Fig. 3:**
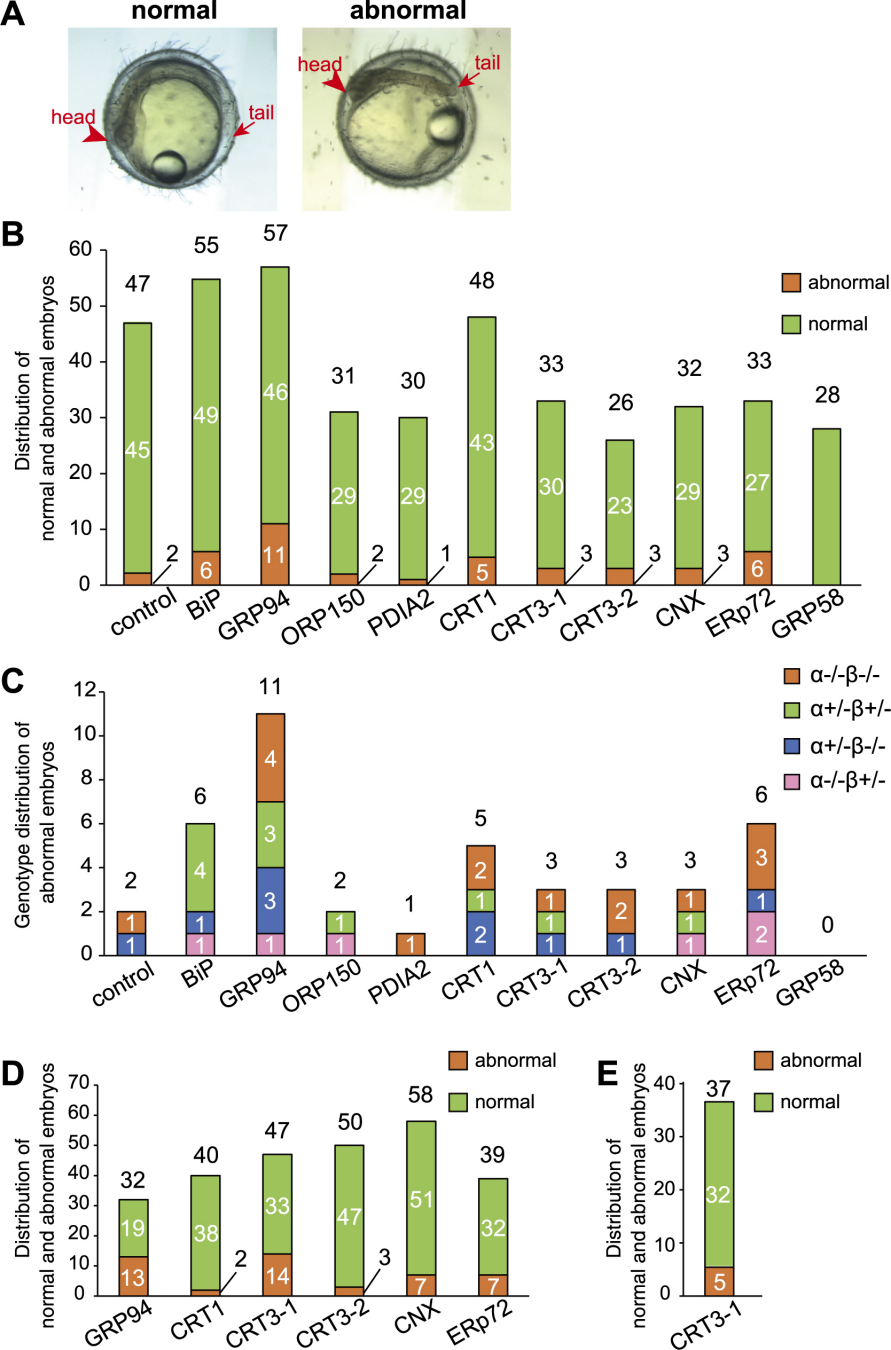
Effect of microinjection of various ER chaperone mRNAs on the morphology of embryos (A) Photograph of normal and abnormal embryos. (B) Distribution of normal and abnormal embryos after microinjection at 100 ng/ml. (C) Genotype distribution of abnormal embryos. (D) Distribution of normal and abnormal embryos after microinjection at 50 ng/ml. (E) Distribution of normal and abnormal embryos after microinjection at 25 ng/ml. These experiments were conducted once with n number indicated in each column.

We microinjected various ER chaperone mRNAs into one-cell stage embryos obtained as in Fig. 1A at the concentration of 100 ng/ml and noticed that some of them showed abnormal phenotypes (developmental impairment) (Fig. 3A) regardless of injected mRNA, except for GRP58 mRNA (Fig. 3B), as well as regardless of genotype (Fig. 3C). Such abnormal phenotypes were also observed when mRNA was microinjected at the concentration of 50 ng/ml (Fig. 3D) and 25 ng/ml (Fig. 3E). Abnormal phenotypes may result from subtle damage to embryos during microinjection. We thus measured the length of the notochord only in embryos with normal phenotypes.

**Fig. 4:**
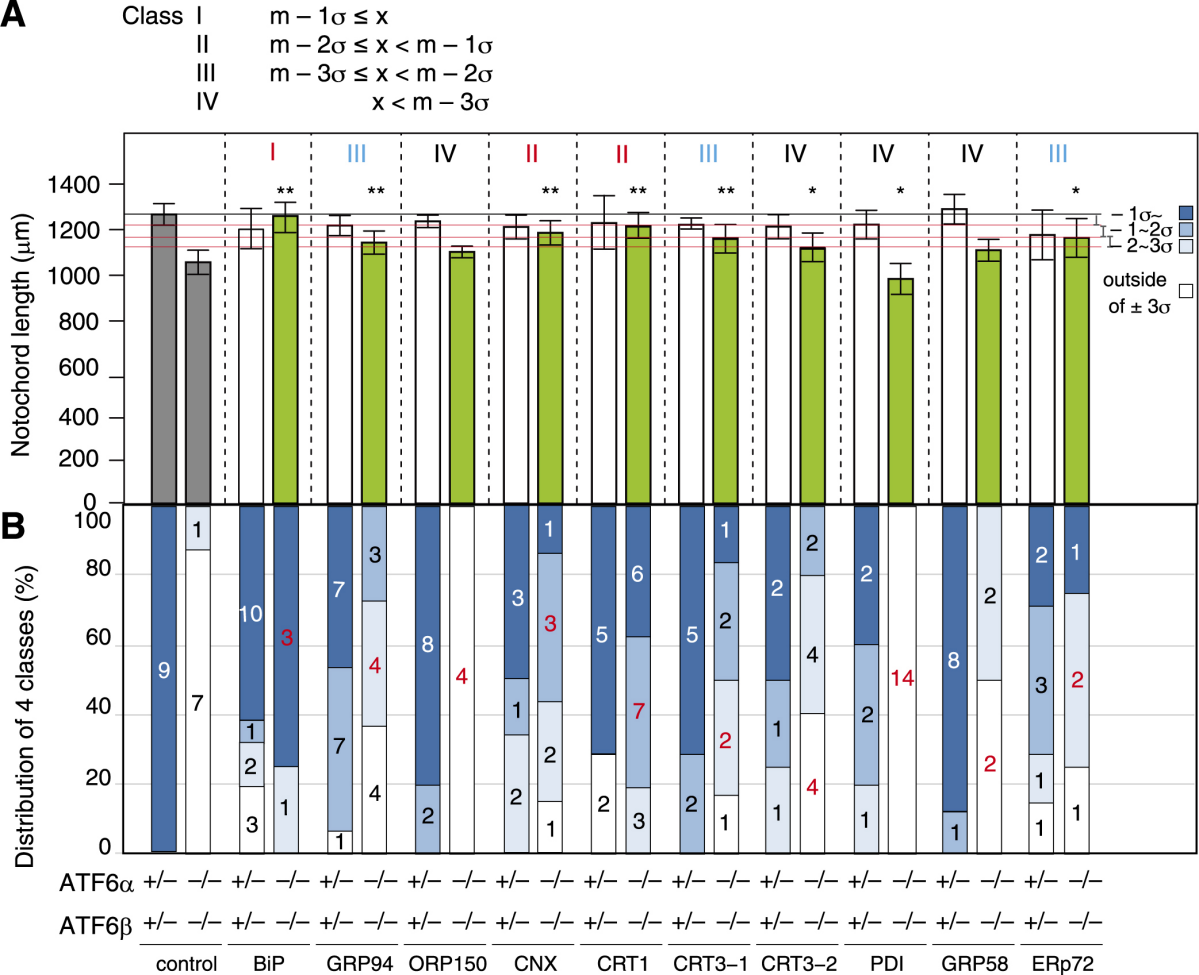
Effect of microinjection of various ER chaperone mRNAs on length of the notochord (A) Notochord length in ATF6α/β-hetero and ATF6α/β-DKO embryos 48 h after non-injection and injection. Asterisks denote the probability of a difference in notochord length between the non-injected and injected ATF6α/β-DKO embryos. (B) Distribution of 4 classes; the red number indicates the representative class. Microinjection of indicated ER chaperone mRNA was separately conducted once with n number indicated in each column.

Forty-eight hours after microinjection of BiP mRNA at the concentration of 100 ng/ml, the notochord in ATF6α/β-DKO embryos [far left green box in Fig. 4A] became significantly longer and similar to that in non-injected ATF6α/β-hetero embryos [far left grey box in Fig. 4A]. As the length of the notochord in non-injected ATF6α/β-hetero embryos is considered to exhibit a normal distribution, we calculated its standard deviation (σ) and categorized the length of the notochord in injected ATF6α/β-DKO embryos into 4 classes: namely class I (dark blue box), m – 1σ ≤ x; class II (middle blue box), m – 2σ ≤ x < m – 1σ; class III (light blue box), m – 3σ ≤ x < m – 2σ; and class IV (white box), x < m – 3σ, where m and x denote the average length of the notochord in non-injected ATF6α/β-hetero embryos and the length of the notochord in injected ATF6α/β-DKO embryos, respectively. Upon microinjection of BiP mRNA, 3 and 1 out of 4 were categorized as class I and class II, respectively [fourth box from the left, Fig. 4B]. We concluded that BiP is a Class I ER chaperone. Based on these criteria, we concluded that CNX and CRT1 belong to Class II; GRP94, CRT3-1 and ERp72 belong to Class III; and ORP150, CRT3-2, PDI, and GRP58 belong to Class IV (Fig. 4).

## Discussion

Here, we conducted qualitative analysis to evaluate the role of ER chaperones in the development of the notochord. We were unable to take account of the differences in the translation efficiency of injected mRNA or the stability of translated protein. Nonetheless, our results suggest that BiP is the first contributor; CNX and CRT1 are the second contributors; and GRP94, CRT3-1, and ERp72 are the third contributors to the development of the notochord. BiP handles newly translocated substrates first among all ER chaperones to push problematic hydrophobic stretches inside of the molecule, so that the molecule can attain the correct three-dimensional structure spontaneously, in accordance with Anfinsen’s dogma (Bukau *et al.*, 2006). Therefore, BiP is considered able to replace other ER chaperones in the development of the notochord if sufficiently overexpressed, which explains the categorization of BiP in class I.

Notably, we previously showed that the expression levels of mRNA coding for BiP, GRP94, CNX, CRT1, CRT2 (currently designated as CRT3-1), CRT3 (currently designated as CRT3-2) and PDI were lower in ATF6α/β-DKO embryos than in ATF6α/β-hetero embryos (Ishikawa *et al.*, 2013). This well-overlapping repertory of ER chaperones identified by two different analyses (ERp72 and PDI are the only exceptions) indicates the importance of these ER chaperones in the development of the notochord.

Genetic analysis in zebrafish showed that the α1 chain of type VIII collagen (Col8a1) (Gansner and Gitlin, 2008), α1 chain of type XV collagen (Col15a1) (Pagnon-Minot *et al.*, 2008), laminin β1 and laminin γ1 (Parsons *et al.*, 2002), and fibrillin-2 (Gansner *et al.*, 2008) are essential to the formation of the notochord. It was previously shown that folding of procollagen is assisted by BiP and GRP94 (Chessler and Byers, 1993; Ferreira *et al.*, 1994), which is consistent with our present results and with the genetic analysis in zebrafish mentioned above. These results explain the categorization of GRP94 in class III.

ERp72 was identified as an ER-resident protein containing three functional thioredoxin-like domains (Mazzarella *et al.*, 1990), and found to be identical to CaBP2 isolated as one of 4 soluble calcium-binding glycoproteins (Van *et al.*, 1993). ERp72 may contribute to calcium homeostasis in the ER (Prins and Michalak, 2011). In addition, it was previously shown that BiP, GRP94, ERp72, and ORP150 form a complex together with a variety of large oligomeric secretory glycoproteins (Kuznetsov *et al.*, 1997). These results may explain the categorization of ERp72 in class III.

Collagens are not *N*-glycosylated whereas laminin and fibrillin are *N*-glycosylated (Fujiwara *et al.*, 1988; Sakai *et al.*, 1986), and are subjected to glycan-dependent productive folding in the ER mediated by CNX/CRT (Ninagawa *et al.*, 2021); CNX/CRT are categorized in class II in this report. We conclude that the CNX/CRT cycle plays a critical role in addition to BiP/GRP94 in the development of the notochord by controlling the quality of glycoproteins.

## Materials and Methods

### Statistics

Statistical analysis was conducted using Student’s t-test, with probability expressed as *p<0.05, **p<0.01 for all figures.

### Fish

Medaka southern strain Cab was used as wild-type fish. Fish were maintained in a recirculating system with a 14:10 hr light:dark cycle at 27.5°C. All experiments were performed in accordance with the guidelines and regulations established by the Animal Research Committee of Kyoto University (approval number: H2819).

### Genotyping

Embryos were suspended in 50 μl of lysis buffer (10 mM NaOH and 0.2 mM EDTA), boiled for 10 min, and then neutralized by the addition of an equal volume of 40 mM Tris-HCl, pH 8.0. DNA fragment containing the mutation site of ATF6α (K149) or ATF6β (S143) was amplified by PCR directly from lysates using the primers 5'-AGTTTTCCCGTCTGCTCATC-3' and 5'-TAACTGCAGTGCGTGCCTAT-3' for ATF6α, and 5'-TACATGTACGGAGACGTGCTG-3' and 5'-GTCTGTGTCTGAATGCTGCTGAT-3' for ATF6β, and the amplified fragments were directly sequenced.

### Cloning of various ER chaperone cDNAs and construction of plasmids

Recombinant DNA techniques were performed according to standard procedures (Sambrook *et al.*, 1989). The integrity of all constructed plasmids was confirmed by extensive sequencing analyses. The various ER chaperone cDNAs obtained were inserted into pCS2-myc-MCS for in vitro transcription. DNA sequences of primers used for cloning and sequencing of various ER chaperones are shown in Table 1.

### Microinjection of mRNA synthesized in vitro into embryo

The 5'-capped BiP mRNA was transcribed in vitro from BiP cDNA obtained previously (Ishikawa *et al.*, 2011) with a mMESSAGE mMACHINE kit (Ambion) and then microinjected into one-cell-stage embryos at the concentration of 100 ng/μl in 0.5× Yamamoto’s buffer, 0.5× I-SceI buffer, and 0.05% phenol red using a FemtoJet (Eppendorf). Microinjection of various ER chaperone mRNAs was conducted similarly.

### Microscopy and notochord length measurement

Brightfield microscopic analysis was conducted using a Leica M205FA stereomicroscope.

Notochord length was determined by analyzing microscopic images using ImageJ (https://imagej.nih.gov/ij/).

## Figures and Tables

**Table 1:** DNA Sequences of primers used for cloning and sequencing

BiP	cloning Fw	5'-TTTATCGATTGAGCAGAGGCGCGAAAGA-3'
	cloning Rv	5'-TTTCCATGGAGGATAACAAATGAGGCCAA-3'
GRP94	cloning Fw	5'-TTTATCGATTCAAGCAGTAACTCAAAATG-3'
	cloning Rv	5'-TTTCCATGGCTTCACTGTTCCACGTTCTC-3'
	sequencing	5'-GAACATGCTCCACATCACTG-3'
	sequencing	5'-CTACTCTGCTTTCCTCGTCG-3'
	sequencing	5'-TTCAAACTTGACGCCCTCCT-3'
ORP150	cloning Fw	5'-TTTATCGATTCCGATACAGAAAGCTCATC-3'
	cloning Rv	5'-TTTCTCGAGTGAAAAATCCACAGTCCTGC-3'
	sequencing	5'-TGCTGGAAGACCCAGTAAGA-3'
	sequencing	5'-AGATCCGAGGTGTTGGGTTT-3'
	sequencing	5'-TACTCAGCAAGGCTTTTAAA-3'
	sequencing	5'-TAAATTTAACTGAGCCTGTC-3'
	sequencing	5'-TTGGCAGCGATTTGCTCCTT-3'
CNX	cloning Fw	5'-TTTATCGATACCGAGCAGCCGAGACCATG-3'
	cloning Rv	5'-TTTCCATGGGGAATCGGACCGACACCTCA-3'
CRT1	cloning Fw	5'-TTTATCGATTTCTCCACGTCGCCACCATG-3'
	cloning Rv	5'-TTTCCATGGCACAGTCTGAGCAGTTTCTG-3'
	sequencing	5'-ACCAGTTCGTGGACAGAACC-3'
CRT3-1	cloning Fw	5'-TTTGGATCCCGGCTGCAGAGCTGAAATTA-3'
	cloning Rv	5'-TTTCCATGGACTACTGCAACCTTTCTTCA-3'
	sequencing	5'-TACAAGGGCAACAATCATCT-3'
CRT3-2	cloning Fw	5'-TTTATCGATTTCTCGAACCTGTTGTGCTT-3'
	cloning Rv	5'-TTTCCATGGGGTTCATCATCAAACTCCTA-3'
PDIA2	cloning Fw	5'-TTTGGATCCCACACAACAACACGAGGATG-3'
	cloning Rv	5'-TTTCTCGAGCTTAGTTTGAACTGTTATCA-3'
	sequencing	5'-ATTGTTCAGTGGTTGAAGCG-3'
GRP58	cloning Fw	5'-TTTATCGATACTTGGTAAGTGGACCAATG-3'
	cloning Rv	5'-TTTCCATGGGTTCCTGTTTGGACGTCTTA-3'
	sequencing	5'-TTTATCGATAGTGGACTGCCCCGTGAGAG-3'
ERp72	cloning Fw	5'-TTTATCGATTTCACGCAGGGAGAACAATG-3'
	cloning Rv	5'-TTTCTCGAGTTTGAAGGAAAACAAAAGTC-3'
	sequencing	5'-CATCAAATGTGAAGATGATG-3'
	sequencing	5'-CACATGGGAGAGCAGGCAGG-3'

## Abbreviations

CNX: calnexin
CRT: calreticulin
DKO: double knockout
hpf: hour post-fertilization
EGFP: enhanced green fluorescent protein
ER: endoplasmic reticulum
KO: knockout
PDI: protein disulfide isomerase
UPR: unfolded protein response
